# Dependence of quantum timescales on symmetry

**DOI:** 10.1016/j.newton.2025.100374

**Published:** 2026-04-06

**Authors:** Fei Guo, Dmitry Usanov, Eduardo B. Guedes, Mauro Fanciulli, Kaishu Kawaguchi, Ryo Mori, Takeshi Kondo, Arnaud Magrez, Michele Puppin, J. Hugo Dil

**Affiliations:** 1 Institute of Physics, École Polytechnique Fédérale de Lausanne, Lausanne 1015, Switzerland; 2 Lausanne Centre for Ultrafast Science (LACUS), École Polytechnique Fédérale de Lausanne, Lausanne 1015, Switzerland; 3 Center for Photon Science, Paul Scherrer Institut, Villigen 5232, Switzerland; 4 CY Cergy Paris Université, CEA, LIDYL, Gif-sur-Yvette 91191, France; 5 Université Paris-Saclay, CEA, LIDYL, Gif-sur-Yvette 91191, France; 6 New Technologies Research Center, University of West Bohemia, Plzen 30100, Czech Republic; 7 Institute for Solid State Physics, The University of Tokyo, Kashiwa, Chiba 277-8581, Japan; 8 Trans-scale Quantum Science Institute, The University of Tokyo, Tokyo 113-0033, Japan; 9 Maryland Quantum Materials Center, Department of Physics, University of Maryland, College Park, MD 20742

**Keywords:** attosecond, photoemission timescale, electron spin polarization, low-dimensional systems, quantum transitions

## Abstract

The role of time in quantum mechanics and the timescale associated with quantum transitions remains an open question in physics, especially on an experimental level. Here we use an experimental method based on spin- and angle-resolved photoemission spectroscopy from spin-degenerate dispersive states to determine the Eisenbud-Wigner-Smith time delay of photoemission. This timescale of the quantum transition is measured for materials with different dimensionality and correlation strength. A direct link is found between the dimensionality, or rather the symmetry, of the system and the attosecond timescale of the quantum transition. The quasi 2-dimensional transition metal dichalcogenides 1T-TiSe_2_ and 1T-TiTe_2_ show timescales around 150 as, whereas in quasi 1-dimensional CuTe, the transition takes more than 200 as. This is in stark contrast with the 26 as obtained for 3-dimensional pure Cu. These results provide new insights into the role of symmetry in quantum timescales and may provide a route to understanding the role of time in quantum mechanics.

## Highlights

• The absolute quantum transition timescale is accessed via the photoelectron spin
• An absolute time duration is experimentally estimated without any time reference
• The attosecond transition timescale increases with reduced dimensionality
• This method yields an alternative to characterize interaction strength in materials

## Accessible overview

The concept of time has troubled philosophers and physicists for thousands of years, and the advent of quantum mechanics has not simplified the problem. The central problem, which can be broken down into several aspects, is the role of time in quantum mechanics. An important open question is the timescale associated with a quantum transition. Energy conservation dictates that such transitions cannot be instantaneous, and their timescale is expected to be in the attosecond (10^−18^ s) range. The challenges of experimentally accessing such timescales lie not only in the fact that they are extremely short but also that using an external timescale risks inducing artifacts. Both challenges can be resolved using quantum interference methods, based on the connection between accumulated phase and time. Here we use the spin of electrons emitted in the photoemission process as an observable of this phase and thus of the timescale of the process itself. These experiments do not require an external reference or clock and yield the timescale required for the wave function to evolve from an initial to a final state at a higher energy upon photon absorption. This time interval is measured for materials with different dimensionality and correlation strength, and a direct link between this dimensionality, or rather symmetry, and the transition timescale is found. Besides yielding fundamental information for understanding what determines the time delay in photoemission, our experimental results provide further insight into what factors influence time on the quantum level and to what extent quantum transitions can be considered instantaneous; they serve as an important piece in the puzzle of time in quantum mechanics.

## Graphical abstract

**Figure:**
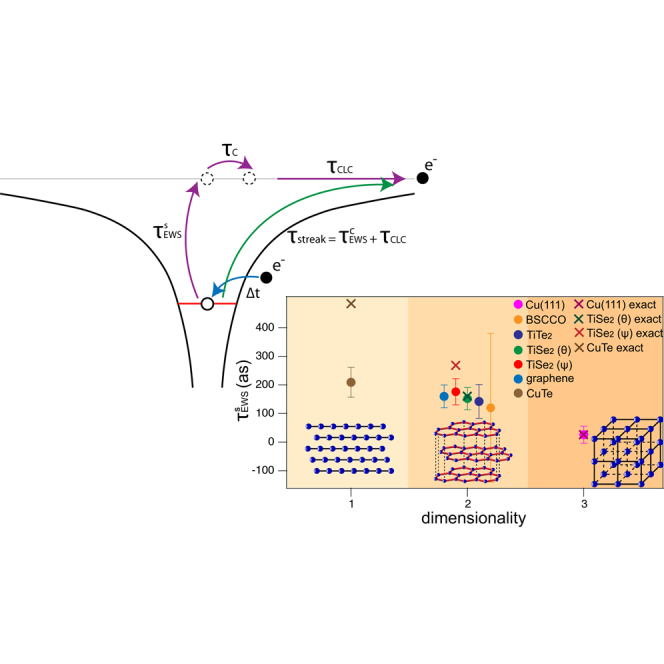

## Teaser

Quantum timescales are notoriously difficult to access in experiments, especially when avoiding projection on an external timescale. Experiments based on spin- and angle-resolved photoemission spectroscopy can help determine the timescale of the quantum transition in photoemission. Guo et al. present such experiments and show an increase in the quantum transition time when going from 3D to quasi-2D and quasi-1D. This indicates an intricate connection between quantum timescales and symmetry.

## Introduction

The role of time in quantum mechanics remains one of the most fundamental problems, despite nearly a century of endeavors. One way to approach this issue is to probe the timescale of quantum processes and their interdependence with other variables. Chronoscopies of tunneling ionization and photoionization, for example, have accessed attosecond timescales close to the atomic unit of time, establishing new limits for accessing fundamental timescales.^1^ In these cases, a bound electron is promoted to the continuum through barrier tunneling in ionization or direct photon-driven transition in photoionization.

While such processes were long regarded as instantaneous down to the femtosecond scale, attosecond spectroscopies have revealed their intrinsic dynamics, reviving the problem of defining time in quantum mechanics.^2^ Improving the measurement precision of attosecond quantum state changes benefits not only applications such as quantum state control^3^ but also fundamental research on relativity tests, and many-body effect probes.^4^^,^^5^ As discussed in more detail below, recent studies have explored alternative routes to direct attosecond-resolved techniques, showing that fundamental timescales can even be determined without any external temporal reference, i.e., without a clock.

In quantum tunneling, several methods have been proposed to measure the time a particle spends inside a barrier, each with limitations. The Wigner phase time compares the positions of tunneled and free particles, but diverges at low tunneling probabilities, leading to unphysical superluminal results.^6^^,^^7^^,^^8^^,^^9^ In the strong-field ionization regime, the attoclock method extracts tunneling times from the scattering angle in elliptically polarized fields,^10^^,^^11^^,^^12^ but results depend heavily on the chosen ionization model.^12^^,^^13^ Larmor clock measurements link dwell time to spin precession in a magnetic field, yet this method has limited applicability to non-rectangular barriers.^6^^,^^14^

A more indirect approach uses interference to access tunneling times. In a Ramsey interferometry scheme, atoms are first prepared in a superposition of two internal states |*g*⟩ and |*e*⟩. After tunneling, the phase difference between the states *arg*⟨*e*_*T*_|*g*_*T*_⟩ = Δ*ω*(*t* + *δt* + *τ*) contains not only the laboratory and relativistic contributions *t* + *δt* but also the tunneling delay *τ*, encoded in the complex part of the tunneling amplitudes *T*_*e*/*g*_.^15^ By comparing this to a reference Ramsey interferometer without a barrier, the tunneling contribution can be isolated. While such a setup has not yet been realized experimentally, similar physics is readily accessible in photoemission.

It comes as an advantage that photoemission has well-understood energetics and kinematics and is widely used in spectroscopy to explore the electronic structure of atoms, molecules, and solids.^16^ Since the 1950s, theory has postulated that photoemission can be seen as a half-scattering process that induces a phase shift in the outgoing wave packet^17^^,^^18^^,^^19^ relative to a wave packet propagating in vacuum.^20^ In the Eisenbud-Wigner-Smith (EWS) model, the half-scattering time delay is related to the phase shift of the scattered wave packet by:

(Equation 1) $$\tau_{\text{EWS}}=\hbar \frac{d\phi }{dE_{k}}\text{,}$$

where *ϕ* is the phase shift, and *E*_*k*_ is the kinetic energy of the outgoing photoelectron.

Attosecond chronoscopy of the photoemission process^11^^,^^21^^,^^22^^,^^23^ has revealed relative delays in the attosecond range between electrons emitted from different states in the same material, and it can thus determine the *relative* EWS time, using as a reference a different electronic state within the same system^2^^,^^24^^,^^25^ or a second reference system.^26^ In order to access the *absolute* EWS time, in analogy to the tunneling amplitude *T*_*e*/*g*_, the phase term *ϕ* of the transition matrix element, assuming the sudden approximation and a free-electron-like final state, $M_{fi}=\langle \psi_{f}|{\hat{H}}_{\mathrm{int}}|\psi_{i}\rangle =Re^{i\phi }$ is required. Phase information is typically lost when probing photoemission intensity; thus, one needs to search for other physical observables that contain explicit information about the phase of the interaction matrix element.

One example of such observables is the angular distribution of photoelectrons from atomic or molecular photoionization,^27^^,^^28^ and it has been demonstrated that the phase term can be extracted by circular dichroism in UV photoelectron diffraction experiments.^29^^,^^30^ However, this method is not practical for crystalline solids, since the small changes in angular distribution are overshadowed by the intrinsic energy-momentum distribution relations of the band structure. In principle, a phase term can also be extracted from interference effects arising from a complex basis, such as that observed in graphene,^31^ and this could be a promising approach.

The spin of photoelectrons emitted at a given angle also depends explicitly on the matrix element phase.^32^^,^^33^ Although initial studies mainly focused on photoionization from atoms in the gas phase, in the case of solids, spin-degenerate initial states result in a spin polarization, which depends on the matrix element phase.^34^ Recently, we developed a semi-analytical model to estimate the EWS photoemission time delay from spin polarization of photoelectrons emitted from solids.^35^ This experimental technique, based on spin- and angle-resolved photoemission spectroscopy (SARPES),^36^ has been used in estimating the EWS time delay for a Cu(111) single crystal and obtained a lower bound of 26 as.^37^

Here, it is worth clarifying the physical meaning of the EWS timescale obtained from spin polarization, the definition of which may vary subtly across communities. In our approach, the spin polarization ***P*** depends on the interference of two partial channels with relative phase shift *ϕ*_*s*_, which leads to the EWS *time delay of the vertical transition*:

(Equation 2) $$\tau_{EWS}^{s}=\hbar \frac{d\phi_{s}}{dE_{k}}\text{,}$$

**Figure 1:**
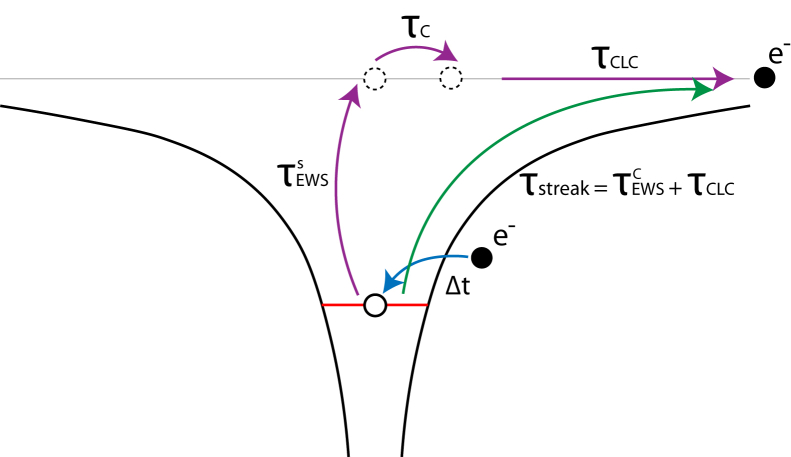
Schematic illustration of the total photoemission time delay Attosecond streaking measures together the time delay of half-scattering and photoelectron transport $\tau_{tot}=\tau_{EWS}^{C}+\tau_{CLC}$ (green arrow), both in the presence of the Coulomb potential. By decomposing the first term further, we have $\tau_{tot}=\tau_{EWS}^{s}+\tau_{C}+\tau_{CLC}$ (purple arrows), where $\tau_{EWS}^{s}$ represents the quantum state transition timescale measured in this work, which is not directly affected by Coulomb interaction. Δ*t* represents the lifetime of the photohole.

The lower bound of which is given by $\left|\tau_{EWS}^{s}\right|\geq \hbar \left|\frac{dP}{dE_{k}}\right|$.^35^ This quantity is conceptually distinct from the streaking timescale, formally defined as $\tau_{streak}=\tau_{EWS}^{C}+\tau_{CLC}$.^1^ There, $\tau_{EWS}^{C}$ represents the half-scattering delay in the presence of the Coulomb potential, while *τ*_*CLC*_ reflects the transport time of the photoelectron. Because of the inclusion of Coulomb interaction, $\tau_{EWS}^{C}$ shows a strong dependence on the photoelectron kinetic energy. In this work, we decompose $\tau_{EWS}^{C}$ further and write $\tau_{streak}=\tau_{EWS}^{s}+\tau_{C}+\tau_{CLC}$. Where *τ*_*C*_ describes the expansion of the wave function and accounts for Coulomb interaction, whereas our main concern is on $\tau_{EWS}^{s}$. We interpret this term as the time associated with the quantum transition itself—the interval required for the wavefunction to evolve from an initial to a final state at a higher energy upon photon absorption, during which multiple photoemission channels interfere and give rise to spin polarization. We note that the timescales described above are distinct from, and not connected to, the photohole lifetime Δ*t*. This separation is schematically illustrated in Figure 1. Importantly, $\tau_{EWS}^{s}$ has been shown to remain nearly invariant across a wide electron kinetic energy range.^37^

**Figure 2:**
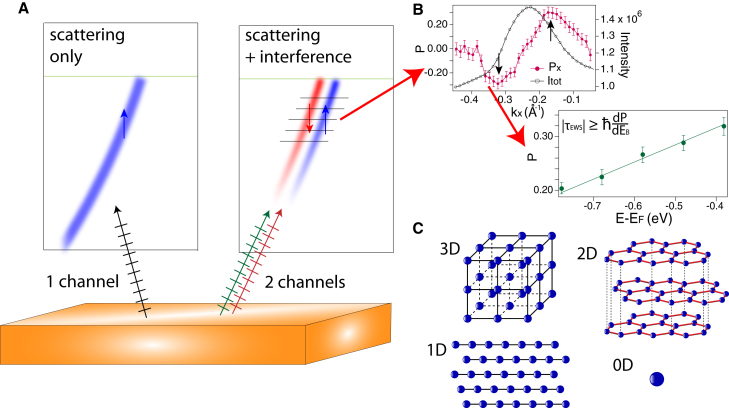
Experimental scheme (A) Schematic drawing of 1-channel and 2-channel photoemission, along with the subsequent single and double polarization feature in spin polarization. (B) Example of measured *P*(*k*) with sign reversal feature (DPF) across the maximum intensity indicated by arrows, and *P*(*E*) constructed from *P*(*k*) at various energies, where the slope is used to calculate $\left|\tau_{EWS}^{s}\right|$. (C) Schematic drawing of matter in 3, 2, 1, and 0 dimensions. Error bars in (B) represent statistical errors based on the total measured intensity.

In order to gain insight into the role of time in quantum mechanics, it is essential to determine what factors influence such fundamental timescales as $\tau_{EWS}^{s}$. In this light, it is interesting to note that $\tau_{EWS}^{s}$ in the high-temperature superconductor Bi_2_Sr_2_CaCu_2_O _8+*δ*_ (BSCCO) was obtained as $\left|\tau_{EWS}^{s}\right|\geq 120\;\text{as}$^38^, much larger than that of Cu(111). It is tempting to link this delay to stronger electronic correlations in BSCCO compared to Cu(111). However, a distinctive and measurable difference also lies in the dimensionality of the crystals and their electronic structure, e.g., BSCCO being quasi-2-dimensional (2D) and Cu being 3-dimensional (3D). Fundamentally, crystal symmetry is embedded in dimensionality, as shown schematically in Figure 2C: an atom is 0D and highly symmetric, and further the level of symmetry reduces from 3D to 1D. In this work, using dimensionality as a simple way of quantifying the degree of symmetry, we extend the study to materials of various dimensionalities and exhibiting different broken symmetry ground states. We focus on two quasi-2D transition metal dichalcogenides: 1T-titanium diselenide (1T-TiSe_2_) with charge-density-wave order, 1T-titanium ditelluride (1T-TiTe_2_) without charge-density-wave order, and a quasi-1D material copper telluride (CuTe), with charge-density-wave order. We find the general trend that $\tau_{EWS}^{s}$ increases with decreasing dimensionality, and thus generally with reduced symmetry.

## Results

### Model to extract EWS time delay

The EWS time delay in photoionization is given by:

(Equation 3) $$\tau_{EWS}=\hbar \frac{d\phi }{dE}=\hbar \frac{d∠\left(\langle \psi_{f}|{\hat{H}}_{\mathrm{int}}|\psi_{i}\rangle \right)}{dE}\text{,}$$

where ∠ draws out the complex phase of the matrix element. In order to make *τ*_*EWS*_ accessible with SARPES, the crucial step is to bridge between $\phi =∠\left(\langle \psi_{f}|{\hat{H}}_{\mathrm{int}}|\psi_{i}\rangle \right)$ and the photoelectron spin. In the picture of electron scattering, because of the spin-orbit coupling term in the scattering potential, the differential scattering cross section is spin-dependent, and an unpolarized electron beam can become spin polarized upon scattering.^33^ The same argument is valid in the case of photoemission: an initially spin-degenerate state can produce spin-polarized photoelectrons even with unpolarized or linearly polarized light that does not carry spin angular momentum,^39^ and the spin polarization was shown to be a result of spin-orbit-induced hybridization of different basis functions representing different single-group spatial symmetries.^40^^,^^41^

To illustrate this concept, we consider the simplest case. When linearly polarized radiation hits the crystal with an off-normal incidence angle, its electric field can be decomposed into *E*_∥_ and *E*_⊥_, which are parallel and perpendicular to the crystal surface, and each of them gives rise to the corresponding complex transition matrix elements *M*_1_ and *M*_2_, respectively. The resulting magnitude of spin polarization of photoelectrons $P=\sum_{i=x,y,z}\hat{i}\;\frac{N_{i}^{↑}-N_{i}^{↓}}{N_{i}^{↑}+N_{i}^{↓}}$ is given by the interference or relative phase difference of these two photoemission channels $P\;\propto \;I\left[M_{1}M_{2}^{*}\right]$. With the magnitude of ***P*** now expressed as a function of the phase shift *ϕ*_*s*_ = *ϕ*_2_–*ϕ*_1_, one can subsequently proceed to estimate the EWS time delay of the vertical transition, and obtain $\left|\tau_{EWS}^{s}\right|\geq \hbar \left|\frac{dP}{dE_{k}}\right|$. It should be noted that the two partial channels do not correspond to separate events, but they together build up the photoelectron wave function. The direction of ***P*** is perpendicular to the reaction plane determined by the incident light and the symmetry of the state under investigation.^40^ This can be used to further refine the obtained timescale in terms of total and scattering time.^35^ However, this requires assumptions that might not hold between different experimental geometries and material systems, and will thus only be used here for a few well-defined cases.

To resume, it is the intrinsic interference of different photoemission channels that gives rise to the observable ***P***, which is analogous to the measured interference signal of the 2 tunneling levels *arg*⟨*e*_*T*_|*g*_*T*_⟩ in the case of the tunneling Ramsey clock.^15^ As elaborated in the methods section and illustrated in Figure 2A, the presence of a Feshbach-type resonance and the consecutive reversal of the spin direction across the band maximum can be used to confirm this intrinsic multi-channel interference. We refer to this as the double polarization feature (DPF) in the measured spin polarization. A similar sign-reversal feature has been observed in the dichroism asymmetry in circular dichroism ARPES, where it is also considered a result of interference effects.^42^

Here, we use the above model to estimate the photoemission timescale from crystalline solids of different dimensionality. With linearly polarized quasi-CW light incident at off-normal angles, we consider spin polarization of photoelectrons from spin-degenerate initial states to arise from interference between the most prominent photoemission channels, which are generally non-zero and assumed to be of the same order of magnitude. With a clear signature of multi-channel interference as shown schematically in Figure 2A, we measure with random order the spin polarization of photoelectrons at different binding energies, construct *P*(*E*), and extract $\left|\tau_{EWS}^{s}\right|\geq \hbar \left|\frac{dP}{dE_{k}}\right|$, as shown in Figure 2B.

### Quasi-2D 1T-TiSe_2_ and 1T-TiTe_2_

To disentangle the possible influence of correlations from dimensionality, we compare the two structurally nearly identical quasi-2D systems 1T-TiSe_2_ and 1T-TiTe_2_. While the former is well known for its charge density wave,^43^^,^^44^ the latter does not show such correlation effects in its bulk form.^45^^,^^46^ Both have been investigated with ARPES,^47^^,^^48^ but to our knowledge, there have been no studies of the electronic band structure with spin resolution and certainly no investigation of the photoemission timescale.

**Figure 3:**
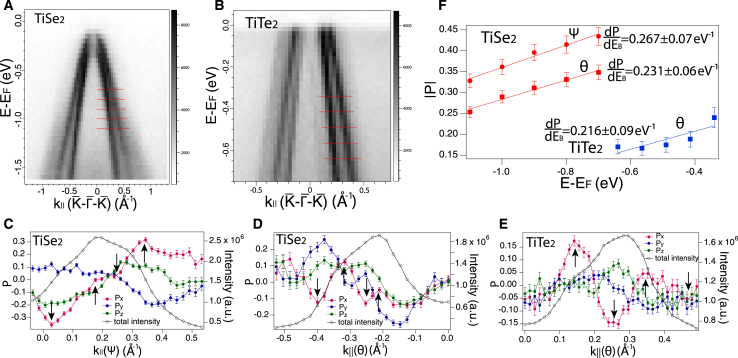
(S)ARPES data for 1T-TiSe_2_ and 1T-TiTe_2_ (A–D) Band maps of (A) 1T-TiSe_2_ and (B) 1T-TiTe_2_ in the $\bar{K}-\bar{\Gamma }-\bar{K}$ direction, taken with *hν* = 67 eV, red lines indicate the binding energies at which momentum distribution curves (MDCs) were taken. MDCs of spin polarization of 1T-TiSe_2_ at *E*-*E*_*F*_ = −0.9 eV in *x*, *y*, and *z* directions obtained by rotating the angle *ψ* (C) and *θ* (D), together with total photoemission intensity. (E) MDCs of spin polarization of 1T-TiTe_2_ at *E*-*E*_*F*_ = −0.49 eV in *x*, *y*, and *z* directions obtained by rotating the angle *θ*. Error bars in (C–E) represent statistical errors based on the total measured intensity, and black arrows indicate 2 pairs of DPFs. (F) Maximum spin polarization magnitude at 5 binding energies extracted from MDCs, resulting in $\left|\tau_{EWS}^{s}\right|\geq 176\pm 46$ as from *ψ*-MDCs of 1T-TiSe_2_, $\left|\tau_{EWS}^{s}\right|\geq 152\pm 39$ as from *θ*-MDCs of 1T-TiSe_2_, and $\left|\tau_{EWS}^{s}\right|\geq 142\pm 59$ as from *θ*-MDCs of 1T-TiTe_2_. Error bars represent data scatter and statistical errors.

Here, we present an investigation of the spin polarization of the photoelectrons from the Se-4*p*_*x*,*y*_ and Te-5*p*_*x*,*y*_-derived valence bands. These show similar band structures along the $\bar{K}-\bar{\Gamma }-\bar{K}$ direction, as illustrated in Figures 3A and 3B. Due to inversion symmetry, these bulk bands are expected to be spin-degenerate. Considering first TiSe_2_, on these bands, 5 indicated binding energies ranging from *E*-*E*_*F*_ = −0.70 to −1.10 eV were chosen at which momentum distribution curves (MDCs) were taken for spin polarization in all spatial directions *x*, *y*, and *z*. These MDCs were taken by respectively scanning the tilt *ψ* (Figure 3C) and polar *θ* (Figure 3D) angles, i.e., by rotating around the sample *x* and *y* axes. Both measurements are along the $\bar{\Gamma }-\bar{K}$ direction in the Brillouin zone, and the sample azimuth was thus rotated by 30° in between.

In the MDCs of ***P*** taken at *E*-*E*_*F*_ = −0.9 eV in Figures 3C and 3D, all 3 components show clear momentum-resolved spin polarization with a maximum value that exceeds ±25% for *P*_*x*_ and *P*_*y*_, respectively. Although the band splitting is hardly distinguishable in the total intensity data, this momentum resolution is recovered in the spin measurement, in which, 2 pairs of overlapping DPFs can be identified from four peaks in spin polarization with alternate signs, each originating from a valence band. These DPFs are indicated by arrows in the respective MDCs. This confirms the presence of multi-channel interference, and we can thus proceed to extract the photoemission timescale.

The magnitude of ***P*** was calculated from all 3 spatial components for the peak around $k=0.33\;{\overset{\circ }{A}}^{-1}$ in Figure 3C, where the vector sum is maximal, and from the peak around $k=-0.4\;{\overset{\circ }{A}}^{-1}$ in Figure 3D, summarized together in red in Figure 3F. Linear fits yield values of $\frac{dP}{dE}$ of 0.267 ± 0.07 eV ^−1^ from *ψ*-MDCs and 0.231 ± 0.06 eV ^−1^ from *θ*-MDCs, respectively. The corresponding minimum estimates for EWS time delays are $\tau_{EWS}^{s}\geq 1.76\pm 0.46\times 10^{-16}$ s = 176 ± 46 as and $\tau_{EWS}^{s}\geq 1.52\pm 0.39\times 10^{-16}$ s = 152 ± 39 as. This similarity in the magnitudes of spin polarization, as well as $\frac{dP}{dE}$ obtained by the 2 measurement geometries indicates that the lower bound of $\tau_{EWS}^{s}$ we extract is not greatly affected by the experimental geometry. However, the spin polarization has noticeably different structures for the two angular directions, i.e., the spin orientation changes with geometry. This is expected, since although the same bands and the same direction in the Brillouin zone are probed, the light polarization is different with respect to the scan direction, possibly resulting in different orbital contributions.^49^

Similar to 1T-TiSe_2_ we measured for 1T-TiTe_2_ *θ*-MDCs at 5 binding energies ranging from *E*-*E*_*F*_ = −0.34 eV to −0.64 eV, as indicated on the band map in Figure 3B. MDCs of ***P*** in all spatial directions taken at *E*-*E*_*F*_ = −0.49*eV* are shown in Figure 3E. Again, spin polarization can be identified in all 3 directions, with the *x* polarization particularly pronounced, showing 2 overlapping DPFs, as indicated by arrows. Using the same method as discussed above, for the peak around $k=0.14\;{\overset{\circ }{A}}^{-1}$, we obtain *P*(*E*) as shown in blue in Figure 3F with the corresponding $\frac{dP}{dE}\;\approx \;0.216\pm 0.09{\text{eV}}^{-1}$ and an estimate of of $\tau_{EWS}^{s}\geq 1.42\pm 0.59\times 10^{-16}$ s = 142 ± 59 as.

The $\tau_{EWS}^{s}$ for 1T-TiTe_2_ is between that obtained for 1T-TiSe_2_ and BSCCO, despite the less strong electronic correlation in the case of 1T-TiTe_2_. Additionally, despite more complex interference effects, a timescale of $\tau_{EWS}^{s}\geq 160$ as has been extracted from spin polarization of the valence band of H-intercalated graphene,^50^ which is clearly 2D. These results are strong indications that dimensionality or symmetry, rather than correlation, plays an important role in the magnitude of $\tau_{EWS}^{s}$. To further verify this hypothesis, we turn to quasi-1D CuTe, which, as typically in this dimension, exhibits CDW order.

### Quasi-1D CuTe

Being quasi-1D and thus susceptible to charge ordering, CuTe has recently been demonstrated to host 3-dimensional CDWs.^51^^,^^52^ The electronic structure of CuTe has been studied with ARPES and time-resolved ARPES, and the quasi-1D states extending along the Γ-Y direction is observed to open a gap below *T*_*CDW*_ = 335 K.^52^^,^^53^^,^^54^ However, the spin polarization of photoelectrons from these bulk-derived quasi-1D states has not been investigated.

**Figure 4:**
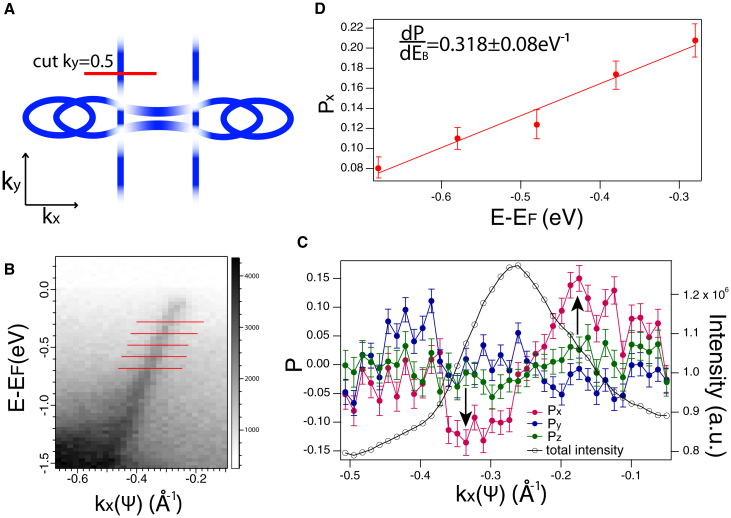
(S)ARPES data for CuTe (A) Schematic drawing of the Fermi surface of CuTe with indication of the constant *k*_*y*_ = 0.5 cut of the band map. (B) Band map of the 1D band taken at *k*_*y*_ = 0.5 and *hν* = 26 eV, red lines indicate the binding energies at which MDCs were taken. (C) MDCs of spin polarization at *E*-*E*_*F*_ = −0.48 eV in *x*, *y*, and *z* directions obtained by rotating the angle *ψ*. Error bars represent statistical errors based on the total measured intensity, and black arrows indicate the DPF. (D) Maximum spin polarization magnitude with each binding energy extracted from *ψ*-MDCs, resulting in $\left|\tau_{EWS}^{s}\right|\geq 209\pm 52$ as. Error bars in (D) represent data scatter and statistical errors.

Figure 4A shows a schematic Fermi surface of CuTe, with the constant *k*_*y*_ cut through the quasi-1D band indicated. Figure 4B shows the corresponding band map, with 5 binding energies ranging from *E*-*E*_*F*_ = −0.28 to −0.68 eV indicated where *ψ*-MDCs of spin polarization are taken. As visible from the MDC at *E*-*E*_*F*_ = 0.48 eV in Figure 4C, spin polarization is most pronounced along the *x* direction reaching ±15%, on which an anti-symmetric DPF is observed, whereas for the other 2 directions, the measured spin polarization is very small within the region of high photoemission intensity. Considering ***P*** in the y and z directions, thus as negligible, the *P*_*x*_ magnitude of the peak around $k_{x}=-0.18\;{\overset{\circ }{A}}^{-1}$ in Figure 4C is plotted for 5 binding energies in Figure 4D. From this we extract a value of $\frac{dP}{dE}\;\approx \;0.318\pm 0.08$ eV ^−1^ and a corresponding lower estimate of the EWS time delay $\tau_{EWS}^{s}>2.094\pm 0.52\times 10^{-16}$ s = 209 ± 52 as. This timescale is significantly larger than that of all other materials investigated thus far, and it confirms the proposed trend in the relationship between EWS timescale and dimensionality, or more generally, with reduced symmetry.

## Discussion

The impact of spatial asymmetry on the EWS time delay has previously been considered by photoionization from gas phase helium with elliptically polarized laser fields^55^ or with a shake-up ionic final state.^56^ For photons with sufficiently high energies, after photoemitting one electron to the ionization continuum, the electron remaining bound to the ion gets excited to one of the ionic Rydberg states, which have much larger spatial extent compared to the deeply bound ground state. This property of the shake-up state makes it strongly polarizable by the laser field, inducing photoionization. During the attosecond timescale of photoionization, the He^+^ ion left behind possesses a finite dipole moment and thus exerts a force on the outgoing photoelectron and causes a retardation. For the ionic Rydberg state *n* = 2, this “correlation time delay” was calculated to be around 6 as. In this context of photoionization, “correlation” is directly proportional to the effective dipole moment of the *He*^+^ ion left behind, or more generally, the extent of asymmetry, and directly affects the total EWS time delay $\tau_{EWS}^{C}$.

**Figure 5:**
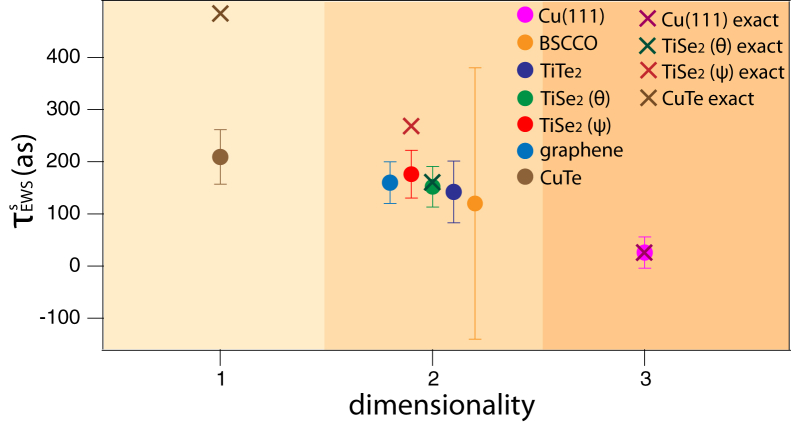
Summary of estimated $\tau_{EWS}^{s}$ vs. dimensionality Dots represent estimated lower limits of $\tau_{EWS}^{s}$, and crosses represent refined estimates taking spin orientation into account. Error bars are obtained from the errors in the linear fits of |*P*| vs. E.

In the context of photoemission from solids, however, the degree of symmetry is manifested in crystal dimensionality as shown in Figure 2C, with a decreasing number of mirror planes from 3D to 1D crystals. Hence, our observation that $\tau_{EWS}^{s}$ is larger for crystals with reduced dimensionality provides a complementary perspective on how reduced symmetry has a significant impact on the ionization timescale, as summarized in Table 1 and in Figure 5. Interestingly, the $\tau_{EWS}^{s}$ obtained from the (quasi-)2D materials appears to directly reflect the expected interlayer coupling in these systems. Further studies are needed to show whether $\tau_{EWS}^{s}$ can indeed be used to quantify the transition in dimensionality.

**Table 1:** Summary of investigated materials with their lower estimated limit of $\tau_{EWS}^{s}$

Material	Dimensionality	$\tau_{EWS,\min}^{s}$ (as)
Cu(111)^37^	3	26 ± 30^a^
BSCCO^38^	2	120 ± 260^a^
TiTe_2_	2	142 ± 59
TiSe_2_-*θ*(*ψ*)	2	152 ± 39(176 ± 46)
graphene^50^	2	160 ± 40^a^
CuTe	1	209 ± 52

For TiSe_2_, results corresponding to the *ψ* geometry are indicated in parentheses.

a The errors were evaluated in this work, using raw data measured by Fanciulli et al.^37^^,^^38^^,^^50^ but not shown in the original publication.

In addition to the lower estimates discussed above, we also attempted to obtain exact values of $\tau_{EWS}^{s}$ for the investigated materials with the direction of ***P***. As described in Fanciulli and Dil,^35^ the angles the spin polarization vector makes with the x*y* plane and *xz* plane determine the exact ratio of the two partial channels, as well as geometrical correction, and lead to a refined estimation. This exact method could only be developed for the *θ*-geometry, which was employed for Cu(111), TiSe_2_, and TiTe_2_. For Cu(111) it yields $\tau_{EWS}^{s}\;\approx \;26$ as and for TiSe_2_, $\tau_{EWS}^{s}\;\approx \;161$ as, while for TiTe_2_ the lower data quality limited calculation reliability. Applying the same formalism to results obtained by the *ψ*-geometry cannot provide reliable absolute values, but the obtained results for TiSe_2_ $\tau_{EWS}^{s}\approx 269$ as and for CuTe $\tau_{EWS}^{s}\;\approx \;485$ as reproduce the same trend of increasing $\tau_{EWS}^{s}$ with reduced symmetry. These refined estimates are also shown in Figure 5. This consistency further supports the robustness of our conclusion.

To summarize, we determined the absolute transition timescale for various materials using a model based on the measured spin polarization of photoelectrons. Although we use the photoemission process in our measurements, the obtained timescales relate to the vertical transition and do not include the ionization or electron emission process itself. A clear trend of increasing timescale with decreasing dimensionality is found, indicating a link between reduced symmetry and the quantum mechanical transition timescale. For a detailed determination of the influence of correlation effects on this timescale, it will therefore be essential to take the dimensionality of the states into consideration, along with other factors such as atomic species and orbital character. Under these conditions, spin-resolved attosecond chronoscopy has the potential to become a tool to characterize the nature and strength of interactions in correlated materials.

Complementary to dimensionality, an interesting potential direction is to investigate the electronic localization and polarizability, which, as the study on helium suggested, could have a direct impact on the EWS time delay. In this regard, Fe-based superconductors, where orbital-selective correlations lead to a coexistence of localized and itinerant Fe-3d states,^57^ could be a promising candidate when probed outside of magnetically ordered phases. Heavy-fermion systems, with their weakly hybridized f-electrons forming narrow quasiparticle bands,^58^ represent an even more localized limit and offer unique opportunities, although with significant experimental challenges. Probing such systems would provide a more stringent test of the emerging link among dimensionality, correlations, polarizability, quantum metrics,^59^ and the photoemission timescale. Furthermore, it will be of interest to explore the influence of rapid changes of the final state and to incorporate the time-reversed low-energy electron diffraction (LEED) state in the model.

Besides yielding fundamental information for understanding what determines the time delay in photoemission, complementary to attosecond streaking and RABBITT experiments, our results provide further insight into what factors influence time on the quantum level, to what extent quantum transitions can be considered instantaneous, and might help understand the role of time in quantum mechanics. Our general approach will be applicable to any quantum transition with a phase-dependent observable. With such methods, it will, for example, be of interest to investigate whether reduced symmetry has a general influence on coherence times in quantum operations. Furthermore, an increase in the duration of a quantum transition can generate additional degrees of freedom for quantum manipulations, such as superposition of states or braiding.

## Methods

1T-TiTe_2_ and 1T-TiSe_2_ single crystals are grown using the chemical vapor transport method with iodine (I_2_) as the transport agent. High-purity Ti and Te or Ti and Se powders are sealed in a quartz ampoule and placed in a two-zone furnace with a controlled temperature gradient. For TiTe_2_, the source is maintained at 800°C and the sink at 625°C, while for TiSe_2_, the temperatures are slightly lower, with the source at 750°C and the sink at 600°C. After a few weeks, millimeter-sized crystals are obtained.

CuTe single crystals are grown using the self-flux method, where high-purity Cu and Te powders are mixed in a non-stoichiometric ratio with an excess of Te as the flux. The mixture is sealed in a quartz ampoule and heated to 600°C to form a homogeneous molten solution. The furnace is then slowly cooled at a controlled rate of 10°C per hour, allowing CuTe crystals to precipitate from the Te-rich melt. After cooling to room temperature, the excess Te flux is removed by sublimation.

SARPES measurements were taken at the COPHEE endstation on the SIS beamline of the Swiss Light Source, Paul Scherrer Institut. Band maps and spin polarization MDCs were measured with *π*-polarized light. Samples were prepared by attaching them to standardized sample holders and attaching a ceramic top post with conductive silver epoxy. Samples were cleaved at ultrahigh vacuum at T = 25 K, and the surface quality was checked with LEED. The measurement geometry is the same as that in Fanciulli et al.^37^, in which, the angle *θ* is scanned by rotating around the sample *y* axis, and the angle *ψ* is scanned by rotating around the sample *x* axis. Spin asymmetry $A=\frac{N_{↑}-N_{↓}}{N_{↑}+N_{↓}}$ was measured by 2 orthogonally positioned classical Mott detectors. Spin polarization was calculated by $P_{i}=\frac{1}{S}A_{i}$ where the Sherman function *S* = 0.08. MDCs were measured for different binding energies in a randomized order to exclude sample aging effects. The chosen range of binding energies is away from hybridization regions, where spurious effects might occur.^60^ The *x*, *y*, and *z* components of spin polarization mentioned in the text are in the sample frame. The zero polarization on all MDCs was calibrated with respect to the general anti-symmetry of the spin texture, and the magnitude of spin polarization was calculated for a single peak for each material.

DPFs of spin polarization were observed in all materials investigated on their initially spin-degenerate bands. This was also seen on dispersive bands of Cu(111), BSCCO, and graphene measured with synchrotron radiation, and was present on 1-step photoemission calculation of Cu(111) conduction band, but absent on core levels of Cu and on dispersive bands of BSCCO measured with *hν* = 6.994 eV.^37^^,^^38^^,^^50^

**Figure 6:**
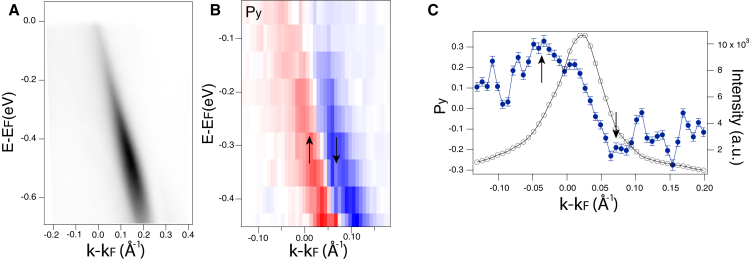
Verification of the double polarization feature (A) Band dispersion of CuTe with *hν* = 6.994 eV. (B) Spin polarization multiplied by the total intensity with quantization axis *y*. (C) *y* spin polarization MDC obtained by moving the electron deflector at *E*-*E*_*F*_ = −0.2 eV. Black arrows indicate 2 opposite peaks in *P*_*y*_. Error bars represent statistical errors based on the total measured intensity.

In order to rule out the possibility that this is a measurement artifact, further spin polarization measurements were performed on a 3D dispersive band of CuTe with *hν* = 6.994 eV at the laser ARPES facility of ISSP, University of Tokyo. The corresponding band map is shown in Figure 6A. With the experimental geometry used, this band shows mainly spin polarization in the *y* direction, with corresponding polarization multiplied by total intensity shown in Figure 6B, and MDC of P_*y*_ at *E*-*E*_*F*_ = −0.2 eV shown in Figure 6C. A sign reversal can clearly be identified across the intensity maximum. This result further confirms the universality of a DPF in spin-degenerate bands. Below, we explain how the presence of a DPF can be used to verify the existence of 2 or more interfering channels, and thus, the model to extract $\tau_{EWS}^{s}$ can indeed be applied.

The DPF can be understood in close analogy to the Feshbach resonance, a well-studied effect in the field of ultracold atomic gases.^61^^,^^62^^,^^63^ A Feshbach resonance occurs when the energy of a scattering state in the continuum approaches that of a bound state, given that the bound state is coupled to the continuum. This effect is typically described with a 2-channel model: one channel represents the bound state and is referred to as “closed”; the other represents the continuum, and is referred to as “open.” In the fully elastic regime, the phase shift *δ* of the continuum wavefunction follows a Breit-Wiger form^64^:

(Equation 4) $$\delta \left(E\right)=\delta_{bg}+\tan^{-1}\left[\frac{\Gamma_{E}}{2\left(E_{res}-E\right)}\right]\text{,}$$

where *δ*_*bg*_ is a slowly varying background, Γ_*E*_ is the resonance width in energy, and *E*_*res*_ is the resonance energy. Across a resonance, as *E*_*res*_-*E* changes sign, the phase shift thus changes sharply by *π*. In the more general inelastic scattering case, multiple channels should be considered, and the total phase shift of all channels ∑_*n*_*δ*_*n*_(*E*) has to follow the above Breit-Wiger form.^65^

While in our model, multi-channel photoemission is assumed, and the EWS time delay can be constructed from a weighted sum^35^:

(Equation 5) $$\tau_{EWS}^{s}\approx \frac{\sum_{q}\hbar \frac{d\delta_{fi}^{q}}{dE}{\left|M_{fi}^{q}\right|}^{2}}{\sum_{q}{\left|M_{fi}^{q}\right|}^{2}}\propto \frac{d\;\sin\left(\phi_{s}\right)}{dE}\propto \frac{dP}{dE}\text{,}$$

where *δ*_*fi*_ = ∠*M*_*fi*_ is the phase of the photoemission matrix element. In the electronic structure of solids, a band is by definition a bound state where such a resonance should occur. Therefore, our observation of a sign reversal of spin polarization across an intensity maximum suggests that dispersive bands with multiple interfering photoemission channels, in analogy to a Feshbach resonance, provides an aggregate phase shift of *π*, and thus a sign reversal of the spin polarization.

## Resource availability

### Lead contact

Requests for further information and resources should be directed to and will be fulfilled by the lead contact, Fei Guo (fei.guo@epfl.ch) and J. Hugo Dil (hugo.dil@epfl.ch).

### Materials availability

This study did not generate new materials.

### Data and code availability

• SARPES data have been deposited at Infoscience under the DOI https://doi.org/10.5075/epfl.20.500.14299/255911 and are publicly available as of the date of publication. All other data reported in this paper will be shared by the lead contact upon request.
• This study did not generate new codes.
• Any additional information required to reanalyze the data reported in this paper is available from the lead contact upon request.

## Acknowledgments

F.G. and J.H.D. acknowledge support from the 10.13039/501100001711Swiss National Science Foundation (SNSF) Project No. 200021-200362. M.F. acknowledge support from the Program ERC CZ, project N. LL2314 (TWISTnSHINE). R.M. acknowledges support from Japan Society for the Promotion of Science (JSPS) KAKENHI Grant No. JP23K13041, The University of Tokyo Edge Capital Partners (UTEC) - University of Tokyo Future Society Initiative (UTokyo FSI) Research Grant Program, and Japan Science and Technology Agency (JST) Precursory Research for Embryonic Science and Technology (PRESTO) Grant No. JPMJPR24LA.

## Author contributions

J.H.D. initiated and designed the research; F.G., D.U., E.B.G., and M.F. performed the experiment; K.K., R.M., and T.K. assisted in data acquisition; A.M. grew the samples; F.G., M.P., M.F., and J.H.D. interpreted the data; M.F. contributed the model; F.G. and J.H.D. wrote the paper with the help of M.P. and M.F.; and all authors commented on the paper.

## Declaration of interests

The authors declare no competing interests.
